# Characteristics of Eyes Requiring Trabeculotomy for Glaucoma With Steroid Treatment: Atopic Dermatitis and Factors Affecting Surgical Outcomes

**DOI:** 10.7759/cureus.47510

**Published:** 2023-10-23

**Authors:** Asako Tanaka, Kenji Suda, Takanori Kameda, Hanako O Ikeda, Masahiro Miyake, Tomoko Hasegawa, Tadamichi Akagi

**Affiliations:** 1 Department of Ophthalmology and Visual Sciences, Kyoto University Graduate School of Medicine, Kyoto, JPN; 2 Division of Ophthalmology and Visual Science, Niigata University Graduate School of Medical and Dental Sciences, Niigata, JPN

**Keywords:** intraocular pressure, trabeculotomy, atopic dermatitis, steroid-induced glaucoma, secondary glaucoma

## Abstract

Purpose: The aim is to analyze the surgical outcomes of glaucomatous patients with steroid treatment and investigate the factors, including atopic dermatitis, associated with the surgical success rate.

Materials and methods: We retrospectively enrolled participants who required first trabeculotomy for glaucoma with steroid treatment between May 2005 and February 2018 and then compared the postoperative outcomes according to the history of atopic dermatitis or surgical procedures. Surgical success was defined as postoperative IOP ≤ 21 mmHg, ≥20% reduction from baseline, and absence of reoperation. The factors influencing the surgical success rates were investigated using mixed-effects Cox regression.

Results: The study included 70 eyes of 46 patients (18 eyes of 12 patients with atopic dermatitis). Postoperative intraocular pressure was not significantly different between eyes with and without atopic dermatitis (12 months after the surgery: patients without atopic dermatitis, 15.4 ± 3.6 mmHg; patients with atopic dermatitis, 16.1 ± 3.9 mmHg; P = 0.65). Twelve months after the surgery, the number of postoperative medications was higher in patients with atopic dermatitis than in those without (2.8 ± 1.3 vs. 2.0 ± 1.7; P = 0.060). However, no significant differences were noted in surgical success rates between patients with atopic dermatitis and those without (*P* = 0.54). Mixed-effects Cox regression of surgical success rate indicated that only the number of preoperative medications significantly influenced surgical success (P = 0.03).

Conclusions: Regardless of the presence of atopic dermatitis, patients taking many preoperative glaucomatous medications might require reoperation.

## Introduction

Steroid-induced glaucoma is a type of secondary open-angle glaucoma characterized by a marked increase in intraocular pressure (IOP) associated with corticosteroid use [1]. Therefore, regular IOP monitoring is recommended when such patients are started on corticosteroids [2,3]. Although steroid discontinuation is the first choice for treatment, it may not be feasible in some patients with specific underlying diseases such as systemic lupus erythematosus or vasculitis [2,3]. In such patients, glaucoma medications (eye drops) are started, and IOP elevation that is resistant to eye drop treatment necessitates glaucoma surgery [4]. Steroid-induced glaucoma responds well to surgical treatment and, if treated appropriately, has a lower risk of blindness than do other types of glaucoma [5]. Nevertheless, in the clinical setting, many patients with steroid-induced glaucoma often experience severe visual field defects [6]. To investigate the clinical characteristics of steroid-induced glaucoma with severe visual impairment, we have collectively reported cases of patients using corticosteroids [7]. Among them, patients with atopic dermatitis visited our hospital with more severe visual field defects at a younger age than did those without atopic dermatitis. Studies have also suggested that glaucoma with atopic dermatitis may be pathologically different from steroid-induced glaucoma and might have underlying causes other than corticosteroid use [8-10].

Many studies have also reported complications of glaucoma surgery and early treatment may not always guarantee a favorable prognosis [11]. Moreover, although many studies to date have reported the outcomes of steroid-induced glaucoma surgery, the surgical methods are gradually changing. Especially, the technique of trabeculotomy is shifting from ab externo to ab interno [12-16]. Trabeculotomy, a surgical procedure to incise trabecular meshwork to create an opening into Schlemm’s canal, is particularly effective for steroid-induced glaucoma [5,17,18]. Although trabeculotomy is often referred to as the first-line treatment, no studies have explicitly investigated history of atopic dermatitis or types of trabeculotomy influence surgical prognosis of glaucoma with steroid treatment.

Therefore, the purpose of this study was to analyze the surgical outcomes of glaucomatous patients with steroid treatment, determine the factors associated with a poor prognosis, identify whether atopic dermatitis is a prognostic factor, determine the time point when glaucoma with steroid treatment should be monitored more carefully after surgery, and evaluate the best surgical methods.

## Materials and methods

This retrospective study adhered to the tenets of the Declaration of Helsinki and was approved by the Institutional Review Board and Ethics Committee of the Kyoto University Graduate School of Medicine (approval number (R2652)).

Patient selection

We retrospectively enrolled patients who underwent their first trabeculotomy at Kyoto University Hospital between May 2005 and February 2018. Eyes with a history of steroid treatment (oral, intravenous, ointment, eye drop, or inhaled administration) were included, as reported in a previous study [7]. However, eyes with a history of uveitis, previous vitreretinal intervention with silicone oil injection or posterior segment steroid implant were excluded from the analysis, because we could not accurately differentiate the causes of IOP elevation. The clinical characteristics and examination results of the patients were collected retrospectively from their medical records. All patients underwent comprehensive ophthalmic examinations, including IOP measurement using a Goldmann applanation tonometer, visual acuity measurement using a Landolt chart at 5 m, slit-lamp examination, gonioscopy, and visual field tests, before the surgery. The reliabilities of the visual field tests (Humphrey Visual Field Analyzer, Carl Zeiss-Meditec, Dublin, CA, USA) using the 24-2 or 30-2 SITA standard testing protocol were defined as a fixation loss < 20% and false-positive and false-negative error rates < 33%). Only reliable results of visual field tests were used in the analyses.

Surgical procedure

In this study, trabeculotomy included both ab interno and ab externo procedures. The surgical technique of ab externo trabeculotomy was as follows: a 4×4 mm^2^ external scleral flap was created; a 3.5×3.5-mm^2^ internal scleral flap was created under the external flap to expose Schlemm’s canal; and two metal probes (trabeculotomes; Handaya Inc, Tokyo, Japan) were inserted into Schlemm’s canal in opposite directions and were rotated by approximately 120° to cut the trabecular meshwork. The internal flap was dissected to accomplish deep sclerectomy. The external scleral flap was closed with 10-0 nylon, and the conjunctiva was closed with an 8-0 absorbable Vicryl suture (Coated VICRYL; Ethicon, Somerville, OH, USA).

Ab interno trabeculotomy was performed in two ways: Trabectome surgery (NeoMedix, Tustin, CA, USA) and suture trabeculotomy (s-LOT) [12,19]. In Trabectome surgery, the microsurgical device handpiece was inserted through a 1.70-mm temporal corneal incision to ablate the nasal trabecular meshwork and the inner wall of Schlemm’s canal to form a 100° to 120° arc. The trabecular meshwork was visualized using a Swan-Jacob lens (Autoclavable Gonioprism; Ocular Instruments, Bellevue, WA, USA) without using any viscoelastic device. S-LOT was performed through a temporal corneal incision using a 25-gauge Microvitreoretinal Blade (Alcon, Fort Worth, TX, USA). The rounded tip of a 5-0 nylon suture was inserted into Schlemm’s canal using the forceps. After passing the suture tip around the circumference of Schlemm’s canal, the suture was pulled out through the same opening.

Definitions of success

Postoperative IOPs and a number of glaucoma medications were assessed by the probability of success in the Kaplan-Meier survival-curve analysis. We defined two success criteria (A and B) based on the guidelines of the World Glaucoma Association: A, absence of reoperation (subsequent glaucoma surgery); B, postoperative IOP ≤ 21 mmHg, ≥20% reduction from baseline, and absence of reoperation. Reoperation was defined as the need for additional glaucoma surgery except for the removal of hyphema in the anterior chamber (anterior chamber irrigation). Patients with IOPs that failed to meet criterion B within three months after the surgery were not considered to have surgical failure. Three months after the first surgery, treatment was determined a failure according to criterion B if IOP was >21 mmHg or <20% reduction from the baseline IOP was observed at two consecutive visits.

Statistical analyses

A generalized estimating equation (GEE) was used to compare the means. Each eye was considered dependent on an individual in the GEE framework. Differences in categorical parameters between the two groups were evaluated using Fisher’s exact tests. Comparisons of success rates between the groups and identification of the relative risk of surgical failure were evaluated using Kaplan-Meier survival analysis and a mixed-effects Cox regression model using the “survival” and “coxme” packages in R. In the mixed-effects Cox regression model, eyes were set nested within patients as a random effect. All P-values presented are two-sided values. The statistical significance was defined as P < 0.05. All analyses were performed using R ver. 3.6.0 (R Foundation for Statistical Computing, Vienna, Austria).

## Results

This study enrolled 70 eyes of 46 patients. Table 1 lists the characteristics of the enrolled patients. The most frequent reason for steroid treatment was atopic dermatitis (18 eyes of 12 patients), followed by systemic lupus erythematosus (seven eyes of four patients). All patients were under oral steroid treatment, except for two patients (two eyes) who received sub-Tenon’s triamcinolone acetonide injection. The surgical procedure was ab externo trabeculotomy in 40 eyes and ab interno in 30 eyes. Postoperative IOPs (at one month, three months, six months, and 12 months after the surgery) were significantly lower than preoperative IOPs at all follow-up periods (P < 0.001). The number of postoperative medications also decreased significantly after the surgery (P < 0.001).

**Table 1:** Clinical characteristics of patients with steroid-induced glaucoma requiring surgery and comparison between patients with and without atopic dermatitis Values are shown as mean ± standard deviation. Comparison is performed using a generalized estimating equation. Criterion A: absence of reoperation (additional glaucoma surgery); Criterion B: postoperative intraocular pressure ≤ 21 mmHg, ≥ 20% reduction from baseline, and absence of reoperation.

	Total (46 patients, 70 eyes)	Patients without atopic dermatitis (34 patients, 52 eyes)	Patients with atopic dermatitis (12 patients, 18 eyes)	P-value
Age at surgery (years old)	48.9 ± 19.6	53.9 ± 18.8	34.4 ± 13.6	<0.001
Sex (female/male)	25/21	16/18	9/3	0.18
Eye (right/left)	37/33	29/23	8/10	0.43
Observation period (total, years)	7.59 ± 7.65	8.28 ± 8.59	5.61 ± 3.27	0.15
Type of trabeculotomy (ab externo trabeculotomy/ ab interno trabeculotomy)	40/30	29/23	11/7	0.79
Single/combined with cataract surgery	48/22	32/20	16/2	0.040
Visual field mean deviation (dB)	-11.82 ± 9.08	-10.48 ± 7.71	-15.39 ± 11.51	0.15
Intraocular pressure (mmHg)				
Preoperative	32.8 ± 8.5	33.0 ± 8.3	32.1 ± 9.4	0.73
Postoperative month 1	16.3 ± 4.6	15.8 ± 3.9	17.8 ± 6.3	0.32
Postoperative month 3	16.3 ± 3.9	16.3 ± 3.6	16.3 ± 4.9	0.99
Postoperative month 6	16.0 ± 3.8	16.2 ± 3.9	15.5 ± 3.5	0.52
Postoperative month 12	15.6 ± 3.7	15.4 ± 3.6	16.1 ± 3.9	0.62
Number of glaucoma medications				
Preoperative	3.5 ± 0.9	3.4 ± 0.9	3.6 ± 0.9	0.53
Postoperative month 1	2.1 ± 1.6	2.0 ± 1.7	2.3 ± 1.4	0.48
Postoperative month 3	2.1 ± 1.6	2.0 ± 1.7	2.5 ± 1.3	0.29
Postoperative month 6	2.2 ± 1.6	2.0 ± 1.7	2.6 ± 1.2	0.24
Postoperative month 12	2.2 ± 1.6	2.0 ± 1.7	2.8 ± 1.3	0.060
Five-year cumulative survival rate (Criterion A, %)	80.0 ± 5.5	83.4 ± 5.8	67.1 ± 13.9	0.51
Five-year cumulative survival rate (Criterion B, %)	76.3 ± 5.8	79.0 ± 6.3	67.1 ± 13.9	0.54

The patients were divided into two groups according to the presence or absence of atopic dermatitis (Table 1). The patients with atopic dermatitis were younger than those with other diseases (P < 0.001) and the number of combined cataract surgeries was smaller in patients with atopic dermatitis than in those without (P = 0.040). Postoperative IOPs (at 1 month, 3 months, 6 months, and 12 months after the surgery) were significantly lower than preoperative IOPs at all follow-up periods in both patient groups (P < 0.001). However, postoperative IOPs were not significantly different between the groups. The mean number of preoperative glaucoma medications decreased significantly after the surgery in both groups (P < 0.05) except for 12 months in patients with atopic dermatitis. Twelve months after the surgery, the number of postoperative medications was also higher with marginal significance in patients with atopic dermatitis than in patients without atopic dermatitis (P = 0.060).

Table 2 shows a comparison between the surgical methods of trabeculotomy. The specific types of trabeculotomy were ab externo in 40 eyes, Trabectome surgery in 17 eyes, and s-LOT in 13 eyes. Postoperative IOPs were significantly lower than preoperative IOPs at all follow-up periods in both surgery groups (P < 0.05). However, no significant differences were observed in preoperative and postoperative mean IOPs between the groups at any follow-up period. The number of postoperative medications in both groups at all postoperative follow-up periods was smaller than the number of medications before the surgery (P < 0.01), but the mean number of medications was significantly different in the two groups at all postoperative follow-up periods.

**Table 2:** Comparison between patients undergoing ab externo and ab interno trabeculotomy Values are shown as mean ± standard deviation. Comparison is performed using a generalized estimating equation. Criterion A: absence of reoperation (additional glaucoma surgery); Criterion B: postoperative intraocular pressure ≤ 21 mmHg, ≥ 20% reduction from baseline, and absence of reoperation

	Ab externo (26 patients, 40 eyes)	Ab interno (21 patients, 30 eyes)	P-value
Age at operation (years old)	45.9 ± 20.5	53.0 ± 17.7	0.22
Sex (female/male)	14/12	11/10	1.00
Eye (right/left)	20/20	17/13	0.63
Atopic dermatitis (-/+)	29/11	23/7	0.79
Observation period (total, years)	9.1 ± 8.7	5.5 ± 5.5	0.12
Single/combined with cataract surgery	31/9	17/13	0.074
Visual field mean deviation (dB)	-12.08 ± 10.12	-11.55 ± 7.99	0.84
Intraocular pressure (mmHg)			
Preoperative	34.2 ± 7.9	30.8 ± 9.0	0.13
Postoperative month 1	17.2 ± 4.9	15.1 ± 4.0	0.09
Postoperative month 3	16.6 ± 3.0	16.0 ± 4.7	0.56
Postoperative month 6	16.5 ± 3.1	15.4 ± 4.5	0.33
Postoperative month 12	16.1 ± 3.0	15.0 ± 4.3	0.22
Number of glaucoma medications			
Preoperative	3.5 ± 1.9	3.3 ± 1.7	0.70
Postoperative month 1	1.3 ± 1.2	3.0 ± 1.6	<0.001
Postoperative month 3	1.5 ± 1.2	3.0 ± 1.7	<0.001
Postoperative month 6	1.5 ± 1.2	3.0 ± 1.7	<0.001
Postoperative month 12	1.7 ± 1.3	2.9 ± 1.7	0.013
Five-year cumulative survival rate (Criterion A, %)	86.5 ± 5.6	66.3 ± 12.3	0.054
Five-year cumulative survival rate (Criterion B, %)	83.3 ± 6.2	62.8 ± 11.9	0.075

Table 3 shows the characteristics of the patients requiring reoperation and those not requiring reoperation. Additional glaucoma surgery was required in 18 eyes. The first operative method in patients requiring reoperation was ab externo trabeculotomy in 11 eyes, Trabectome surgery in six eyes, and s-LOT in one eye. IOPs at one and three months after the surgery were higher in patients with reoperation than in patients without reoperation. The mean number of postoperative glaucoma medications was higher in patients with reoperation than in patients without reoperation at all follow-up periods. However, no significant differences were noted in reoperation rates between patients with atopic dermatitis and those without atopic dermatitis (P = 1.00).

**Table 3:** Characteristics of patients requiring reoperation Values are shown as mean ± standard deviation. Comparison is performed using a generalized estimating equation. BGI: Baerveldt glaucoma implant; IOP: intraocular pressure; Criterion A: absence of reoperation (additional glaucoma surgery); Criterion B: postoperative intraocular pressure ≤ 21 mmHg, ≥ 20% reduction from baseline, and absence of reoperation.

	Patients without reoperation (34 patients, 52 eyes)	Patients with reoperation (12 patients, 18 eyes)	P-value
Age at operation (years old)	49.4 ± 20.2	47.4 ± 17.9	0.74
Sex (female/male)	14/20	7/5	0.34
Eye (right/left)	29/23	8/10	0.43
Atopic dermatitis (-/+)	39/13	13/5	1.00
Observation period (total, years)	7.90 ± 8.27	6.73 ± 5.58	0.53
Type of trabeculotomy (ab externo trabeculotomy/ ab interno trabeculotomy)	29/23	11/7	0.79
Single/combined with cataract surgery	35/17	13/5	0.78
Visual field mean deviation (dB)	-11.74 ± 9.53	-12.09 ± 7.81	0.89
Intraocular pressure (mmHg)			
Preoperative	32.4 ± 8.8	33.9 ± 7.8	0.50
Postoperative month 1	15.3 ± 3.7	19.4 ± 5.8	0.023
Postoperative month 3	15.4 ± 3.1	19.3 ± 4.7	0.003
Postoperative month 6	15.6 ± 3.6	17.3 ± 4.2	0.22
Postoperative month 12	15.2 ± 3.0	16.9 ± 5.1	0.25
Number of glaucoma medications			
Preoperative	3.4 ± 0.9	3.53 ± 0.92	0.76
Postoperative month 1	1.8 ± 1.6	2.9 ± 1.3	0.008
Postoperative month 3	1.8 ± 1.6	3.3 ± 1.0	<0.001
Postoperative month 6	1.8 ± 1.6	3.3 ± 1.2	<0.001
Postoperative month 12	1.8 ± 1.5	3.2 ± 1.4	0.004
Reoperation mode (ab externo trabeculotomy/ ab interno trabeculotomy /trabeculectomy/BGI)	-	3/7/7/1	-
Five-year cumulative survival rate (Criterion A, %)	100	38.9 ± 11.5	<0.001
Five-year cumulative survival rate (Criterion B, %)	93.9 ± 4.2	38.9 ± 11.5	<0.001

**Figure 1:**
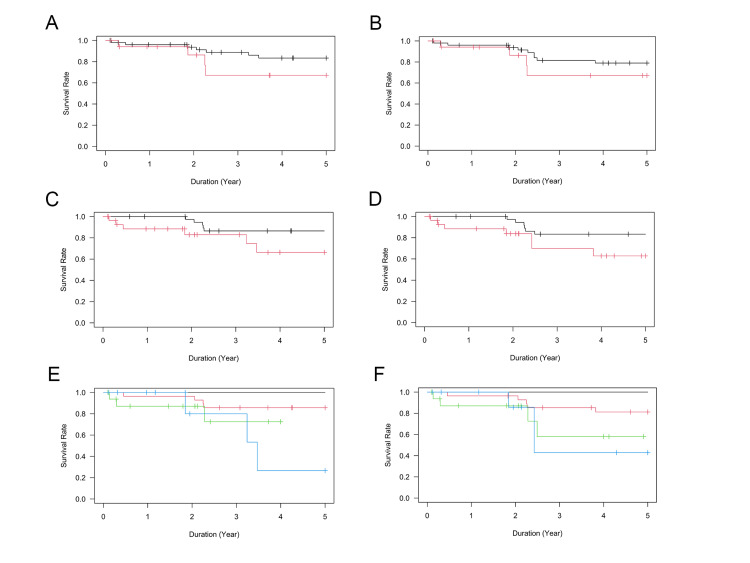
Results of Kaplan–Meier survival analyses of surgical outcome Kaplan–Meier cumulative survival curves showing comparisons of reoperation rates (criterion A; A, C, E) and success rates (criterion B; B, D, F). (A, B) Comparisons between patients without atopic dermatitis (black line) and those with atopic dermatitis (red line), (C, D) comparisons between ab externo group (black line) and ab interno group (red line), and (E, F) comparisons according to the number of preoperative glaucoma medications (black: two or fewer medications; red: three medications; green: four medications; blue: five medications).

The results of the Kaplan-Meier cumulative survival analyses are shown in Figures 1A-1F. No significant difference in survival was found between patients with and without atopic dermatitis under criteria A and B. The five-year cumulative survival rate under criterion A was 83.4 ± 5.8% in patients without atopic dermatitis and 67.1 ± 13.9% in patients with atopic dermatitis (P = 0.51; Table 1, Figure 1A). As for criterion B, the five-year cumulative survival rate was 79.0 ± 6.3% in patients without atopic dermatitis and 67.1 ± 13.9% in patients with atopic dermatitis (P = 0.54; Table 1, Figure 1B). A marginally significant difference was observed between the types of trabeculotomy under criteria A and B. The five-year cumulative survival rate under criterion A was 86.5 ± 5.6% in the trabeculotomy Ab externo group and 66.3 ± 12.3% in the filtration surgery Ab interno group (P = 0.054; Table 2, Figure 1C). As for criterion B, the five-year cumulative survival rate was 83.3 ± 6.2% in the trabeculotomy Ab externo group and 62.8 ± 11.9% in the filtration surgery Ab interno group (P = 0.075; Table 2, Figure 1D). Mixed-effects Cox regression analyses indicated that only the number of preoperative medications significantly influenced the surgical success rate of glaucoma surgery (Table 4). Indeed, the survival rates after reoperation differed significantly according to the number of preoperative medications used (P < 0.001; Figures 1E, 1F).

**Table 4:** Mixed-effects Cox regression models influencing the success rates of glaucoma surgery for steroid-induced glaucoma CI: confidence interval; MD: mean deviation determined using the Humphrey Visual Field Analyzer; Criterion A: absence of reoperation (additional glaucoma surgery); Criterion B: postoperative intraocular pressure ≤ 21 mmHg, ≥ 20% reduction from baseline, and absence of reoperation.

	Hazard ratio [95% CI], P-value			
	Criterion A		Criterion B	
	Univariate	Multivariate	Univariate	Multivariate
Age (year)	0.991 [0.948 to 1.04], 0.70	0.981 [-0.138 to 0.0996], 0.75	0.998 [0.949 to 1.05], 0.95	0.997 [-0.0833 to 0.0770], 0.94
Sex (male = 1)	0.717 [0.129 to 3.98], 0.70	0.234 [-4.93 to 2.03], 0.41	0.879 [0.12 to 6.42], 0.90	0.574 [-2.87 to 1.75], 0.64
Atopic dermatitis	1.87 [0.293 to 12.0], 0.51	0.821 [-4.72 to 4.33], 0.93	2.00 [0.221 to 18.1], 0.54	1.50 [-2.63 to 3.44], 0.79
Intraocular pressure (mmHg)	1.00 [0.927 to 1.08], 0.99	1.04 [-0.0747 to 0.146], 0.53	0.971 [0.898 to 1.05], 0.46	0.975 [-0.115 to 0.0653], 0.59
Preoperative MD	0.997 [0.919 to 1.08], 0.94	1.04 [-0.0639 to 0.139], 0.47	0.997 [0.919 to 1.08], 0.95	0.993 [-0.0910 to 0.0769], 0.87
Number of preoperative glaucoma medications	5.32 [1.32 to 21.3], 0.02	7.03 [0.285 to 3.62], 0.02	5.14 [1.24 to 21.4], 0.02	3.37 [0.113 to 2.32], 0.03

Table 5 shows the postoperative adverse events. During the follow-up, postoperative complications did not differ significantly in any comparison.

**Table 5:** Postoperative adverse events

	Total	Patients without atopic dermatitis	Patients with atopic dermatitis	P-value	Ab externo trabeculotomy	Ab interno trabeculotomy	P-value	Patients without reoperation	Patients with reoperation	P-value
Hyphema (-/+) (eyes)	57/21	37/15	12/6	0.77	31/9	18/12	0.13	37/15	12/6	0.77
Intraocular pressure spike > 30 mmHg (-/+) (eyes)	66/12	44/8	16/2	1.00	36/4	24/6	0.31	47/5	13/5	0.11
Transient hypotony < 5 mmHg (-/+) (eyes)	72/6	50/2	16/2	0.27	37/3	29/1	0.63	49/3	17/1	1.00

## Discussion

In our study, we found that atopic dermatitis emerged as the most prevalent underlying condition necessitating surgery in glaucomatous patients undergoing steroid treatment. On comparing the characteristics between patients with atopic dermatitis and those with other reasons for steroid use, we found no significant differences in preoperative and postoperative IOP or success rates. Although different criteria were used in the survival analyses, the survival rate of patients with atopic dermatitis showed a similar tendency to that of patients reported in a previous study [17], which reveals that the survival rate was not significantly different, but the 80% survival time was shorter in patients with atopic dermatitis. Interestingly, our study also showed that the 80% survival time until reoperation for patients with atopic dermatitis was about two years, and the number of reoperations decreased after two years of surgery. Moreover, the number of glaucoma medications at 1 year after the surgery was higher in patients with atopic dermatitis with marginal significance and the results of Mixed-effects Cox regression revealed an association of the number of glaucoma medications with the surgical success. These indicate the importance of diligent follow-up, especially for two years after the surgery in atopic patients with many glaucoma medications. In addition, the reason why the higher number of glaucoma medications at one year postoperatively in patients with atopic dermatitis is partly because the number of combined cataract surgeries was smaller in patients with atopic dermatitis. Combined cataract surgery with trabeculotomy may positively influence IOP reduction [20]. However, the above result could also be because of the therapeutic resistance to glaucoma surgery in patients with atopic dermatitis as discussed below.

On comparing the success rates of surgical procedures, we found a marginally significant difference between ab externo and ab interno. Several previous studies have reported the surgical outcomes of trabeculotomy in patients with steroid-induced glaucoma. Honjo et al. reported that trabeculotomy could be a useful and effective surgical treatment for patients with steroid-induced glaucoma, and it helped maintain IOP at ≤21 mmHg [5]. Ngai et al. reported that the 12-month survival rate after Trabectome surgery was 93% [21]. Dang et al. found that Trabectome surgery resulted in greater IOP reduction in patients with steroid-induced glaucoma and higher baseline IOPs than in controls with primary open-angle glaucoma [22]. However, Kinoshita-Nakano et al. reported better prognoses in patients with open-angle glaucoma undergoing trabeculotomy rather than Trabectome surgery, and the survival rates were worse with Trabectome surgery than with ab externo trabeculotomy [14]. Although ab interno trabeculotomy is inferior to ab externo trabeculotomy in terms of IOP reduction, considering ab interno trabeculotomy keeps the conjunctiva intact, it may be a better choice to preserve the conjunctiva for trabeculectomy in the future. Similarly, in cases where tube shunt surgery may have to be performed instead of trabeculectomy, surgeons must take precautions to avoid exposing the tube from a weak conjunctiva in patients with atopic dermatitis [11].

In the current study, reoperation (additional glaucoma surgery) was required in 18 eyes, and trabeculotomy was selected as the surgical procedure for reoperation in seven eyes. When trabeculotomy is performed as the first surgery, surgeons have the option of making an incision as wide as possible using s-LOT; however, in young patients in particular, the surgeons have the option to repeat trabeculotomy multiple times to avoid trabeculectomy as far as possible. Sato and Kawaji performed a randomized controlled study comparing the surgical outcomes between the extents and locations of ab interno trabeculotomy and found that the extent of trabeculotomy did not affect surgical success [23]. If a certain extent of intact trabecular meshwork is left, the surgical procedure for reoperation can be determined according to the residual visual field as with the first surgery. However, the surgical outcomes in a young cohort reported by Chen et al. suggested that circumferential trabeculotomy produced more favorable results than other types of trabeculotomy in terms of IOP control [24]. Nevertheless, the extent of s-LOT in the first surgery warrants further investigation in the future.

With respect to reoperation rates, multivariate analysis in the current study shows that the higher the number of preoperative glaucoma medications, the earlier the need for reoperation. In our study, postoperative IOP and the number of glaucoma medications were also higher in patients requiring reoperation at all follow-up periods. Detailed reports on the change in the number of glaucoma medications before and after surgery have been scarce. However, a randomized controlled trial conducted as part of the Laser in Glaucoma and ocular HyperTension study suggested that selective laser trabeculoplasty (SLT) had better therapeutic effects on IOP reduction and fewer side effects when used as the first-line treatment rather than after the introduction of many glaucoma medications [25,26]. This indicated that the ability of aqueous outflow through the trabecular meshwork may be reduced by long-term and multiple topical medications. On the other hand, real-world evidence from the UK showed that the success rate of SLT was not associated with the number of medications or disease severity [27]. In fact, our results also showed that the number of medications did not significantly affect surgical success (criterion B) (according to the results of Table 4). Despite these contradictory findings, we must remember that IOP may not decrease after the first surgery and that reoperation may be required for patients under maximally tolerated medical treatment before surgery.

This study has some limitations owing to its retrospective and single-center study design. The sample size, consisting of 70 eyes from 46 patients, is relatively small. We focused on the outcome of the first surgery and did not analyze the outcome of reoperation or the prognosis after reoperation. All the subjects in this study had a history of steroid treatment, but we did not limit to what extent the steroid treatment was associated with ocular hypertension or the development and progression of glaucoma. Moreover, atopic glaucoma can develop in the eyes with steroid treatment, thus the subjects in this study may not be called “steroid-induced” glaucoma. The selection of surgical procedures was contingent upon the discretion of the operating surgeon, owing to the retrospective nature of the study, potentially introducing an additional source of bias. Notably, glaucoma implant surgery may be predominantly considered in cases involving patients with atopic conditions. The determination of surgical indications and the evaluation of outcomes associated with glaucoma implants in atopic patients represent important avenues for future research.

## Conclusions

Reoperation rates did not differ between patients with atopic dermatitis and those with other reasons for steroid use, but it is noteworthy that one year after surgery, patients with atopic dermatitis experienced an increase in the number of glaucoma medications. In addition, the greater the number of preoperative medications, the lower the success rate of trabeculotomy. Careful monitoring taking into account the findings of this study can aid in early detection and intervention, ultimately improving the long-term management of glaucoma in this population.
